# Numerical Simulation of the Behavior of Hydrogen Source in a Novel Welding Process to Reduce Diffusible Hydrogen

**DOI:** 10.3390/ma13071619

**Published:** 2020-04-01

**Authors:** Shinichi Tashiro, Naoki Mukai, Yoshihide Inoue, Anthony B. Murphy, Tetsuo Suga, Manabu Tanaka

**Affiliations:** 1 Joining and Welding Research Institute, Osaka University, Osaka 5670047, Japan; suga@jwri.osaka-u.ac.jp (T.S.); tanaka@jwri.osaka-u.ac.jp (M.T.); 2 Kobe Steel, Ltd., Kanagawa 251-8551, Japan; mukai.naoki@kobelco.com (N.M.); inoue.yoshihide@kobelco.com (Y.I.); 3 CSIRO Manufacturing, Lindfield, NSW 2070, Australia; tony.murphy@csiro.au

**Keywords:** cold cracking, diffusible hydrogen, FCAW, low-hydrogen welding process, welding torch, suction, numerical simulation

## Abstract

This study aims to reduce the diffusible hydrogen content in deposited metal during gas metal arc welding (GMAW) and flux-cored arc welding (FCAW) which induces cold cracking. To achieve this, a novel welding torch with a dual gas nozzle has been developed. This special welding torch decreases the hydrogen source gas evaporated from a welding wire by the suction from the inner gas nozzle. In order to improve the suction efficiency of this evaporated gas, precise control of the suction gas flow is indispensable. In this paper, a simplified numerical simulation model of this process has been described. This model can take account of the evaporation of the hydrogen source gas from the wire while simulating the behavior of the shielding gas and the arc. Using this model, the effect of suction nozzle structure and torch operating conditions on suction gas flow pattern and suction efficiency was also investigated to understand the process mechanism. Furthermore, the diffusible hydrogen content in deposited metal was measured by chromatography as a validation step. Results show that some of the shielding gas introduced from a shielding nozzle was drawn inward and also branched into an upward flow that was sucked into the suction nozzle and a downward flow to a base metal. This branching height was defined as the suction limit height, which decisively governed the suction efficiency. As a result, in order to reduce the diffusible hydrogen, it was suggested that the suction limit height should be controlled towards below the wire position, where the evaporation rate of the hydrogen source gas peaks through optimization of the suction nozzle design and the torch operating conditions.

## 1. Introduction

In order to meet the demand for higher efficiency in various industries, the size of structures such as buildings is increasing day by day. Generally, in order to improve the strength and rigidity of the components, the approach taken is to increase the thickness and strength of the steel material. However, the use of these materials leads to problems, such as difficulty in controlling the welding process due to cold cracking.

The causes of cold cracking are generally related to four factors: base metal composition, diffusible hydrogen, residual stress and thermal history [1,2]. There are several technologies for preventing cold cracking. 

For example, as a material technology, a Thermo-Mechanical Control Process (TMCP)-type high-tensile steel has been developed and is now widely used. This material can prevent a hardened structure occurring in a weld heat-affected zone. Other methods often involve diffusible hydrogen. The most common method is to preheat and postheat the work piece to increase the diffusivity of hydrogen in the steel and to release it from the base metal and weld metal. However, heat management such as preheating and postheating requires a great deal of workload and energy consumption, and there are also serious safety and health issues such as working in high temperature. Therefore, attention is focused on the diffusible hydrogen content in deposited metal. Hereinafter, the diffusible hydrogen content in deposited metal is referred to as the diffusible hydrogen content for omission. Decreasing this diffusible hydrogen will also aid in to lighten the heat management, as a measure for welding construction management of large structures.

For these reasons, much work has been carried out to reduce the diffusible hydrogen content for many years. The most basic technique to reduce the diffusible hydrogen content is to select low-hydrogen consumables and apply appropriate storage conditions [3]. Optimization is possible depending on welding parameters. For example, Kawabe et al. measured the diffusible hydrogen content of flux-cored arc welding (FCAW) under a constant welding current of 270 A with varying contact tip-to-work distances (CTWDs) [4]. Further, they found that the diffusible hydrogen content decreased with an increase in CTWDs despite the increased wire feeding speed (WFS). In cases with long CTWDs, a large part of the hydrogen source gas is considered to evaporate from a higher wire position due to enhanced wire temperature by Joule heating. Thus, the great part of this gas spreads out with shielding gas to the surroundings without entering the weld pool. Mukai et al. reported the diffusible hydrogen content increased almost in proportion to welding current under a constant CTWD, which is thought to be caused by an increase in WFS [5]. The relevance of the effect of main welding parameters on the diffusible hydrogen content was summarized by Fydrych et al. [6]. These results show that the diffusible hydrogen content can be reduced to some extent by optimizing welding parameters. However, in general, this optimization is severely limited due to the requirement to obtain a sound weld bead. Moreover, in order to prevent cold cracking, a more dramatic reduction of the diffusible hydrogen content is expected.

There are also many studies on improving welding materials, the oldest being on covered electrodes. Hydrogen reduction methods include raw material selection and treatment technology to reduce the amount of water (water-containing minerals, etc.) in the flux, and design technology to reduce the hydrogen partial pressure in the arc atmosphere with a gas-generating agent (without hydrogen) [7,8]. In order to reduce hydrogen in flux-cored wire, production technology for wire annealing in the manufacturing process is being studied. In this case, one paper is reported. The paper reports that the diffusible hydrogen content can be reduced to the level of 0.5 ml/100 g [9]. It was also reported that the diffusible hydrogen was considerably reduced by adding yttrium or neodymium as ferro powders in the metal core of welding consumables [10]. Retained austenite in the weldment also acts as hydrogen trap and reduces the diffusible hydrogen content [11]. The diffusible hydrogen can be manipulated through flux modification, which can be brought on by fluoride additions in the cored flux [12]. The paper concludes that a careful addition of oxidizing ingredients such as fluoride or calcite reduces the diffusible hydrogen.

Aside from welding materials, a welding method to reduce the diffusible hydrogen content using a special shielding gas has been studied [13], and it is reported that the diffusible hydrogen content in the weld metal is reduced by mixing CF_4_ in the shielding gas. However, there are still many issues to be considered, such as the safety of the above gas, instability of the arc, etc.

As a method for reducing the diffusible hydrogen content by using welding equipment, a novel welding torch with a dual gas nozzle, which enables suctioning the hydrogen source gas evaporated from a wire, has been developed. This torch was based on the results of the study on the evaporation of moisture and other hydrogen sources as well as its penetration route to the weld pool. Then, the feasibility of reducing the diffusible hydrogen content during gas metal arc welding (GMAW) and FCAW was discussed [4,14]. As a result, this torch was found to be effective for both GMAW and FCAW. Furthermore, in GMAW of high-tensile thick plates, cold cracking was successfully prevented [15]. In order to improve the suction efficiency of this welding torch, the design and operating conditions must be optimized based on a deep understanding of the process mechanism. In the previous paper [16], as a first step of the research, ignoring the evaporation of the hydrogen source gas from the wire, basic parametric investigation on suction gas flow pattern was carried out through numerical simulation. In this paper, a simplified numerical simulation model of this process has been described. This model can take account of the evaporation of the hydrogen source gas from the wire while simulating the behavior of the shielding gas and the arc. Using this model, the effect of suction nozzle structure and torch operating conditions on suction gas flow pattern and suction efficiency was mainly investigated to understand the process mechanism. The process mechanism is thought to be basically similar to GMAW and FCAW, and so only FCAW is addressed in this paper.

## 2. Simulation Model

### 2.1. Arc Model

We have developed a 3D numerical simulation model of FCAW using a novel torch to reduce the diffusible hydrogen content. In this model, the grade of the hydrogen source gas is assumed to be H_2_O. This arc model is coupled with a simplified evaporation model of H_2_O from the wire described in the next subsection. The arc model is conventionally used in the arc welding field [17]. Here, three levels of the suction nozzle length, the shielding gas flow rate and the suction gas rate are calculated for a parametric study. 

**Figure 1:**
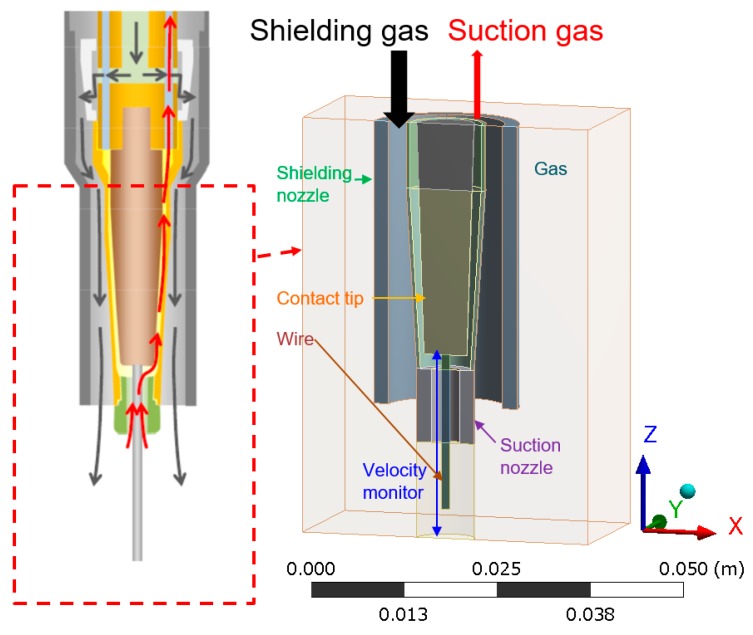
Schematic illustration of the simulation region.

Figure 1 shows a 3D simulation region with a width *X* of 40.0 mm, a length *Y* of 20.0 mm and a height *Z* of 56.5 mm, which consists from the region of a wire, a contact tip, a suction nozzle, a shielding nozzle and a gas. In order to reduce the computational load, a plane symmetrical region is assumed, and only one side of the region is calculated. The mesh size is non-uniform and approximately 0.1 mm at a minimum. The wire diameter is 1.2 mm and the contact tip-to-work distance (CTWD) is 25.0 mm. The inner diameter of the suction nozzle is 4.0 mm. The length of the suction nozzle is defined as the distance between the contact tip and the tip of the suction nozzle. The standard length is 12.0 mm. Two types of length are calculated—4.5 or 14.5 mm—against the standard length. The inner diameter of the shielding nozzle is 16.0 mm. The arc length is 4.0 mm. The contact tip region, the suction gas outlet region, the suction nozzle region, the shielding gas inlet region, the shielding nozzle region and the gas region are defined on the top boundary. The base metal surface is defined on the bottom boundary. The inside of the base metal is not considered in this simulation. The gas region is defined on the side boundary. The polarity and welding current is DC+ and 280 A, respectively. The shielding gas is CO_2_ and its flow rates are 15, 25 or 35 l/min, with 25 l/min as the standard condition. The suction rates are 3, 5 or 10 l/min, with 5 l/min as the standard condition. The suction rate of 0 corresponding to the conventional process was unavoidably omitted due to the difficulty in converging the calculations. In the initial condition, the simulation region is filled with nitrogen gas-simulating air in order to investigate effect of the gas suction on shielding effect. Based on the obtained distribution of nitrogen mole fraction, the shielding effect can be evaluated. Table 1 shows the simulation conditions.

In this simulation, distributions of the temperature, flow, CO_2_ mole fraction, etc., are obtained by solving the following equations assuming steady state.

Mass conservation:

(1) $$\nabla \cdot \left(\rho \vec{u}\right)=S_{H2O}$$

Momentum conservation:

(2) $$\nabla \cdot \left(\rho \vec{u}\vec{u}\right)=-\nabla p+\nabla \cdot \vec{\vec{\tau }}+\rho \vec{g}+\vec{j}\times \vec{B}$$

Energy conservation:

(3) $$\nabla \cdot \left(\rho h\vec{u}\right)=\nabla \cdot \left(k\nabla T\right)+\vec{j}\cdot \vec{E}-R_{loss}$$

Mass conservation of CO_2_:

(4) $$\nabla \cdot \left(\rho Y_{CO2}\vec{u}\right)=\nabla \cdot \left(\rho D_{CO2}\nabla Y_{CO2}\right)$$

Mass conservation of H_2_O:

(5) $$\nabla \cdot \left(\rho Y_{H2O}\vec{u}\right)=\nabla \cdot \left(\rho D_{H2O}\nabla Y_{H2O}\right)+S_{H2O}$$

Current conservation:

(6) ∇·σ∇Φ=0

Ohm’s law:

(7) $$\vec{j}=-\sigma \nabla \Phi =\sigma \vec{E}$$

Vector potential:

(8) $$\nabla^{2}\vec{A}=-\mu_{0}\vec{j}$$

Magnetic field:

(9) $$\vec{B}=\nabla \times \vec{A}$$

where *ρ* is the mass density, $\vec{u}$ is the velocity, *S*_H2O_ is the source term relating to H_2_O evaporation from the wire, *p* is the pressure, $\vec{\vec{\tau }}$ is the viscosity, $\vec{g}$ is the gravity, $\vec{j}$ is the current density, $\vec{B}$ is the magnetic field, *h* is the enthalpy, *k* is the thermal conductivity, *T* is the temperature, $\vec{E}$ is the electric field, *R*_loss_ is the arc radiation loss, *Y*_CO2_ is the CO_2_ mass fraction, *D*_CO2_ is the diffusion coefficient of CO_2_ in N_2_, *Y*_H2O_ is the H_2_O mass fraction, *D*_H2O_ is the diffusion coefficient of H_2_O in CO_2_, *σ* is the electrical conductivity, *Φ* is the electric potential, $\vec{A}$ is the vector potential, and $\mu_{0}$ is the permeability of vacuum. The diffusion coefficients are obtained by solving the viscosity approximation equation [18].

Table 2 shows the boundary conditions. The gauge pressure of the outer boundary in contact with the gas region is 0. The specified mass flow rates to the suction gas outlet and the shielding gas inlet are given. The temperature of 300 K on the outer boundary is given. The constant temperature of 300 K inside the contact tip is set for simplicity. In the wire, the temperature is 300 K just below the contact tip and 1800 K at the wire tip, and the temperature rises linearly from the contact tip to the wire tip. On the boundaries in contact with the gas region except for the shielding gas inlet, two types of conditions are given. The gradient of mass fraction of CO_2_ and H_2_O is 0 where the gas flows out, and the mass fraction of CO_2_ and H_2_O is 0 where the gas flows in. The mass fraction of CO_2_ is 1 and that of H_2_O is 0 on the shielding gas inlet. The current is given inside the contact tip region on the top boundary and conducts to the bottom boundary, where the potential is set to 0. The vector potential is 0 on the side boundary and the gradient of potential is 0 on the top and bottom boundaries.

The thermodynamic and transport properties of the arc as functions of temperature and CO_2_ mole fraction are calculated [19,20,21] under the local thermodynamic equilibrium assumption [22]. The effect of the contamination of H_2_O into CO_2_ or N_2_ on the thermodynamic and transport properties is ignored, because the maximum mole fraction of H_2_O is only below 10^−3^% as presented in Section 3. Metal vapor evaporation from a droplet and metal transfer are not considered for simplicity. The calculation is carried out using ANSYS Fluent 18.1 (Ansys, Inc., Canonsburg, PA, USA).

### 2.2. Simplified Evaporation Model of the Hydrogen Source Gas

The model in simulation described the evaporation process of H_2_O, which is considered as the major hydrogen source gas in a flux-cored wire. H_2_O is thought to be mainly contained in fluxes. The evaporation process is very complex and is extremely difficult to model accurately. Here, the source term, *S*_H2O_, in Equation (1) and in Equation (5), is assumed to be obtained taking into account the evaporation rate per unit volume, *R*_eva_, being the Arrhenius type function as presented in Equation (10). *S*_H2O_ is determined so that the volume integration of *R*_eva_ inside the wire at a certain height agrees with the area integration of evaporation flux on the wire surface calculated from *S*_H2O_.

(10) $$R_{eva}=A\exp(-\frac{E_{a}}{RT_{w}})\times Y_{H2OW}\times M_{wire}$$

where A is the constant, E_a_ is the activation energy (mole evaporation enthalpy at 100 ℃: 40.7 kJ/mol), R is the gas constant, *T*_w_ is the wire temperature, *Y*_H2OW_ is H_2_O content (mass fraction) in the wire, and *M*_wire_ is the mass density of the wire material. The constant A is determined taking into account experimental measurement of the diffusible hydrogen content as follows.

Equation (11) is a mass conservation equation of H_2_O in the wire. $\vec{u_{wire}}$ is the wire feeding speed. The source term, *S*_H2OW_, related to the H_2_O consumption in the wire by evaporation is also calculated form *R*_eva_. *S*_H2OW_ is only given to cells adjacent to the surface. *D*_H2OW_ is the diffusion coefficient virtually defined for providing H_2_O around the wire center to the wire surface.

(11) $$\nabla \cdot \left(\rho Y_{H2OW}\vec{u_{wire}}\right)=\nabla \cdot \left(\rho D_{H2OW}\nabla Y_{H2OW}\right)-S_{H2OW}$$

The H_2_O content is set to 400 ppm at the bottom of the contact tip, which is a typical value of flux-cored wire. H_2_O in the wire is transported to the wire tip end at a wire feeding speed of 15.0 m/min, and its content is gradually decreased due to the evaporation.

**Figure 2:**
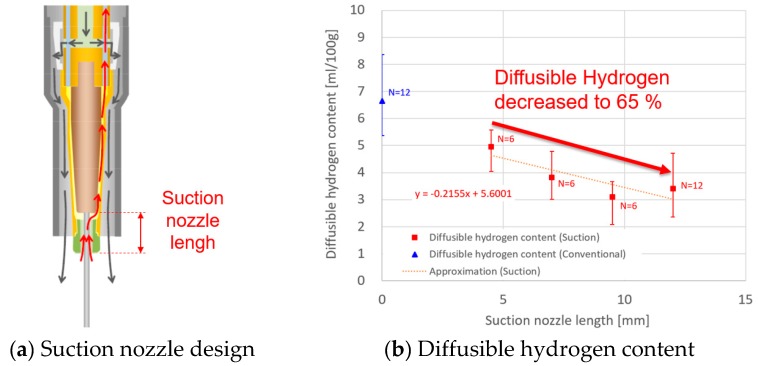
Diffusible hydrogen content measurement: (**a**) suction nozzle design; (**b**) diffusible hydrogen content measured as a function of contact tip-to-suction nozzle distance.

In order to determine A, the diffusible hydrogen content was experimentally measured as a function of the suction nozzle length. The experimental method is described in Section 3. Figure 2 shows the suction nozzle design and the measured diffusible hydrogen content. The result for suction nozzle lengths of 4.5, 7.0, 9.5 and 12.0 mm was presented together with that for conventional torch. For the former, an approximated straight line is also presented due to large variations in the plot. As the suction nozzle length increased, the diffusible hydrogen content decreased almost linearly. The straight line shows that the diffusible hydrogen content at 12.0 mm decreases to approximately 65% of that at 4.5 mm. From the comparison with the diffusible hydrogen content in the case of the conventional torch, the suction was found to enable effectively reducing the diffusible hydrogen content. 

**Figure 3:**
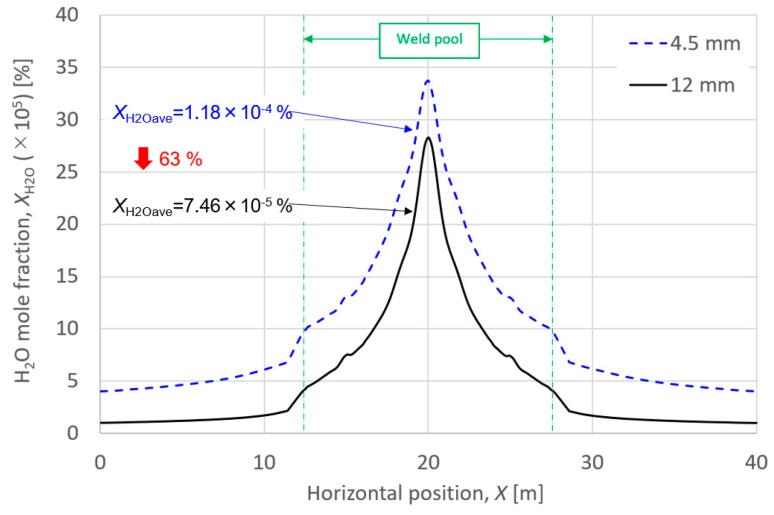
Horizontal distribution of the H_2_O mole fraction over base metal surface.

Figure 3 shows the horizontal distribution of the H_2_O mole fraction over base metal surface, when A is 10,000. The results for suction nozzle lengths of 4.5 and 12.0 mm were compared. The weld pool width observed in the experiment was approximately 15 mm. It is assumed that the average value of the H_2_O mole fraction over the weld pool surface, which is defined as *X*_H2Oave_, is reflected in the amount of hydrogen absorbed in the weld pool and is finally correlated with the diffusible hydrogen content. From the comparison between two cases, when the suction nozzle length was extended from 4.5 to 12.0 mm, the average value of the H_2_O mole fraction over the weld pool surface decreased to 63%, which almost correlated with the decrease in the diffusible hydrogen content of 65% obtained through the experiment. Therefore, an A of 10,000 was determined to be used in this calculation. Details of the above calculation results are given in Section 4.

**Figure 4:**
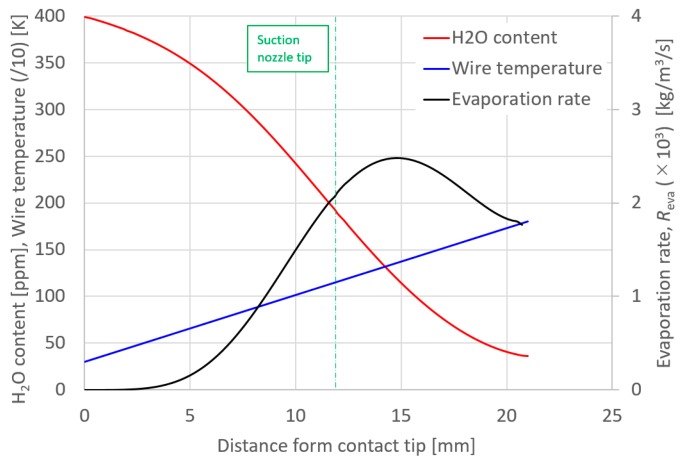
Axial distribution of the H_2_O content in wire, wire temperature and H_2_O evaporation rate.

Finally, Figure 4 shows the axial distribution of the H_2_O content in the wire, wire temperature and H_2_O evaporation rate. As described above, it is assumed that the wire temperature rises linearly from the contact tip towards the wire tip. The evaporation rate, *R*_eva,_ increased with wire temperature and the H_2_O content gradually decreased due to evaporation. Following this, the evaporation rate decreased below the peak at a distance of 15 mm from the contact tip. At the wire tip, H_2_O content became 40 ppm, but this was finally emitted from the wire tip to the arc.

As described above, since it is extremely difficult to model the H_2_O evaporation process from the wire strictly, the evaporation model was constructed under some assumptions.

## 3. Diffusible Hydrogen Content Measurement

The amount of the diffusible hydrogen content in the weld metal was measured by the gas chromatography method according to JIS Z 3118 (2007). This measuring method has good correlation with the mercury method. However, there are different opinions about the constant of proportionality. It has been said that the same level of measurement results can be obtained by both methods [23]. JIS Z 3118 is based on this point. A comprehensive review of several other reports suggests that the value of JIS Z 3118 is approximately 0.7 times larger than the measured value of the mercury method [24,25,26]. In any case, however, it was considered to be effective as an experimental value used for relative comparison with the simulation. The outline of measurement is as follows.

### 3.1. Preparation of Measurement Samples

A test plate (JIS G 3106 (2015) SM400B (400 MPa class steel): 12 mm × 25 mm × 40 mm) and two end tabs (12 mm × 25 mm × 45 mm) that were subjected to hydrogen removal treatment in advance were welded. End tabs were arranged at both ends of the test plate so that the start and end of welding do not affect the measurement. After welding, the test plate was quenched in iced water and stored in dry ice-saturated alcohol solution after removing the end tabs.

### 3.2. Collection of Diffusible Hydrogen

The prepared measurement sample was inserted into a dedicated collection vessel and held at 45 °C for 72 hours to release diffusible hydrogen from the inside of the sample into the vessel.

### 3.3. Measurement of Amount of Hydrogen

The collection vessel was connected to a measuring device, and hydrogen was separated and determined by a column while flowing Ar was used as a carrier gas. The determined amount of hydrogen was converted to the amount per mass of the deposited metal measured from the weight change in the test plate and treated as the amount of the diffusible hydrogen content in the weld metal.

In this test, a welding wire with a diameter of 1.2 mm was applied according to JIS Z 3313 T 49J 0 T1-1 C A-U (Flux-cored wire for 490 MPa class steel). Welding was carried out by changing parameters as in the above-mentioned simulation. The arc voltage was 32 V and the welding speed was 350 mm/min, and both were kept constant. This measurement was performed at least three times under each condition.

## 4. Results and Discussion

**Figure 5:**
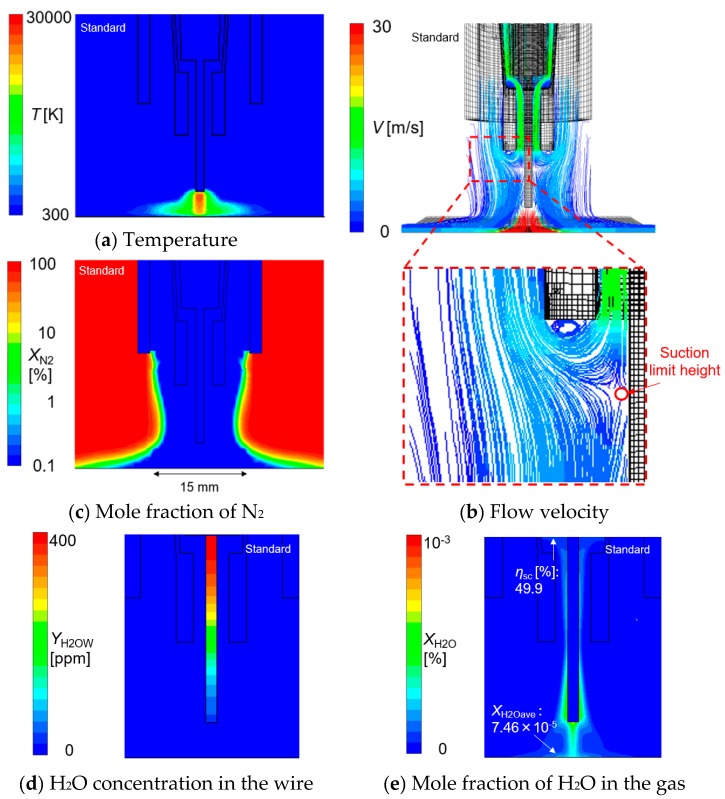
An example of the results in the standard condition: (**a**) temperature, (**b**) flow velocity and (**c**) mole fraction of N_2_, (**d**) H_2_O concentration in the wire and (**e**) mole fraction of H_2_O in the gas.

Figure 5 shows 2D distributions of temperature, flow velocity and the mole fraction of nitrogen on the symmetry plane in the standard condition. 

Figure 5a indicated that the arc temperature reached approximately 30,000 K at the maximum around the wire tip due to strong Joule heating by the high current density. The metal vapor evaporation was not considered in this simulation as explained above. When the metal vapor is considered, it is said that the arc temperature will become lower, because the current flow path is thought to expand by the change of the thermophysical property of arc plasma due to mixing of the metal vapor and shielding gas [27].

Figure 5b shows path lines of the gas flow colored by the flow velocity overwritten on the mesh of the torch and base metal. Although the flow velocity increased up to 200 m/s near the wire tip due to a large Lorenz force, the maximum value of the scale range was set to 30 m/s because the flow velocity around the suction nozzle tip was especially important. Some of the shielding gas introduced from the shielding nozzle was drawn inward and branched into an upward flow that was sucked into the suction nozzle and a downward flow to the base metal approximately 3 mm below the suction nozzle. This branching height is defined as the suction limit height.

Figure 5c shows the distribution of nitrogen mole fraction. The blue region shows a region where the CO_2_ mole fraction is high, and the shielding effect is good. However, this region slightly narrows towards the center when approaching from the shielding nozzle exit to the base metal. The weld pool, with width of approximately 15 mm, was covered with the shielding gas. Furthermore, nitrogen penetration into the weld pool during arc welding especially dominates in the region where molecules are dissociated by high-temperature arc plasma [27,28]. Nitrogen dissociation occurs above approximately 4000 K [29]. In this model, which does not consider the metal vapor, this high temperature range corresponded to a diameter of 12 mm. Since the nitrogen mole fraction was sufficiently low in the above region, the effect of gas suction from the nozzle on the shielding effect was thought to be very small.

Figure 5d shows the H_2_O concentration in the wire. H_2_O decreased from 400 ppm at the bottom of the contact tip with decreasing height.

Figure 5e shows the mole fraction of H_2_O in the gas. *η*_sc_ is the suction efficiency obtained by dividing the suction rate of H_2_O by the total evaporation rate of H_2_O. The total evaporation rate is defined as area integration of the evaporation flux on the wire surface. The result indicated that almost half of the H_2_O evaporated from the wire was sucked by the suction nozzle in the standard condition. 

**Figure 6:**
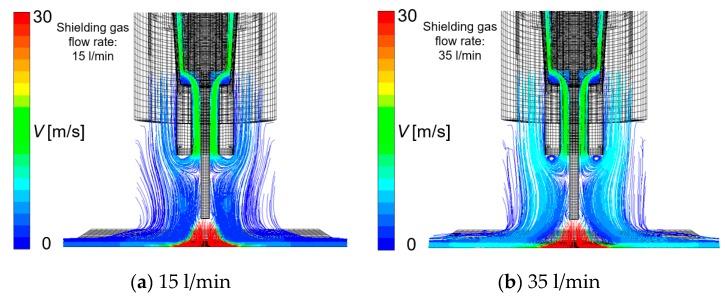
The effect of the shielding gas flow rate on gas flow velocity: (**a**) 15 l/min, (**b**) 35 l/min.

**Figure 7:**
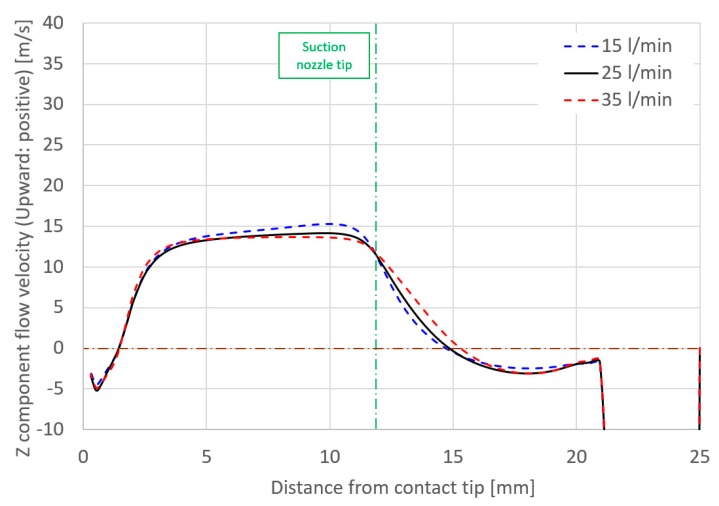
Axial distribution of *Z* component flow velocity as a function of the shielding gas flow rate.

**Figure 8:**
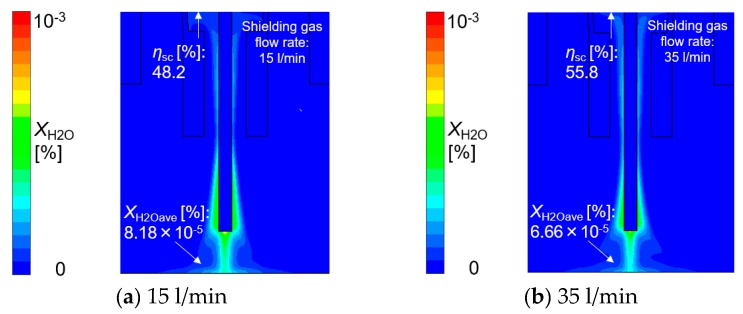
The effect of the shielding gas flow rate on the mole fraction of H_2_O: (**a**) 15 l/min, (**b**) 35 l/min.

**Figure 9:**
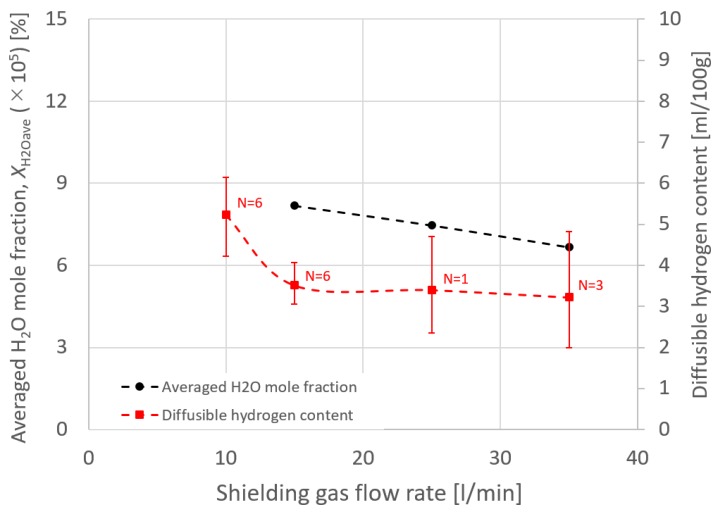
The measured diffusible hydrogen content and the averaged H_2_O mole fraction as a function of the shielding gas flow rate.

Considering the effect of torch operating conditions on distributions of the suction gas flow and the mole fraction of H_2_O, simulations are shown in Figure 6, Figure 7, Figure 8 and Figure 9. These figures show the effect of the shielding gas flow rate. The results at 15 and 35 l/min are presented in Figure 6 and Figure 8. Figure 7 shows a 1D distribution of *Z* component flow velocity along the line expressed as “velocity monitor” in Figure 1, where positive and negative values correspond to upward and downward velocities, respectively. A change in the sign of the velocity was seen at a distance of approximately 15 mm from the contact tip, corresponding to the suction limit height. It is expected that the lower suction limit height enables sucking the H_2_O evaporated from the wire into a wider region, which can contribute to the reduction of the diffusible hydrogen content. The gas flow velocity in the vicinity of the shielding nozzle and the suction nozzle increased with the shielding gas flow rate. It was also found that the suction limit height became lower with the increase in the shielding gas flow rate. Figure 8 shows that with a higher shielding gas flow rate of 35 l/min, *η*_sc_ was 55.8%, which was higher than 48.2% at 15 l/min. As a result of higher *η*_sc_, *X*_H2Oave_ became lower. From this result, the evaluation based on the suction limit height defined here is considered to be appropriate. Figure 9 shows the measured diffusible hydrogen content and *X*_H2Oave_ as a function of the shielding gas flow rate. The diffusible hydrogen content was found to decrease with the shielding gas flow rate, which was in line with the tendency of change in *X*_H2Oave_ in the simulation result.

**Figure 10:**
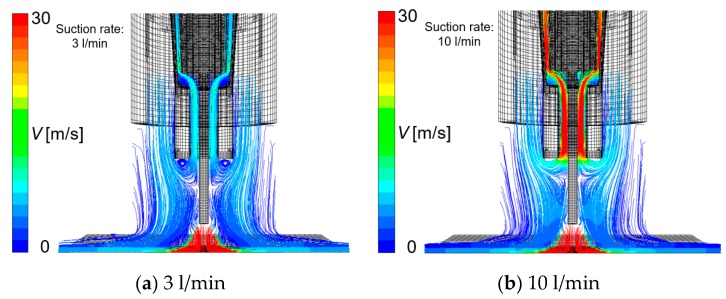
The effect of the suction rate on gas flow velocity: (**a**) 3 l/min, (**b**) 10 l/min.

**Figure 11:**
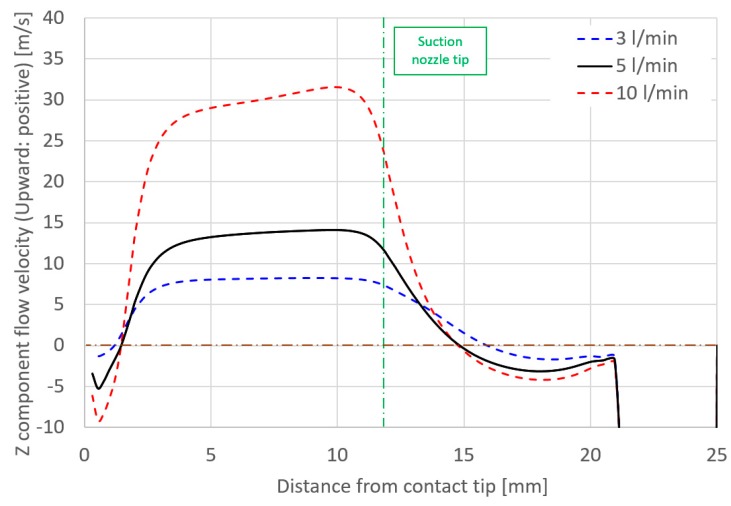
Axial distribution of *Z* component flow velocity as a function of the suction rate.

**Figure 12:**
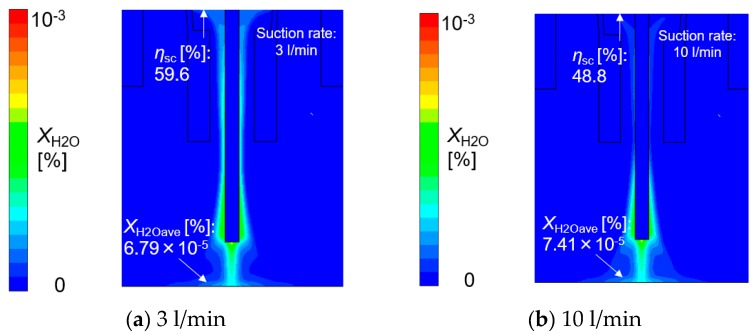
The effect of the suction rate on the mole fraction of H_2_O: (**a**) 3 l/min, (**b**) 10 l/min.

**Figure 13:**
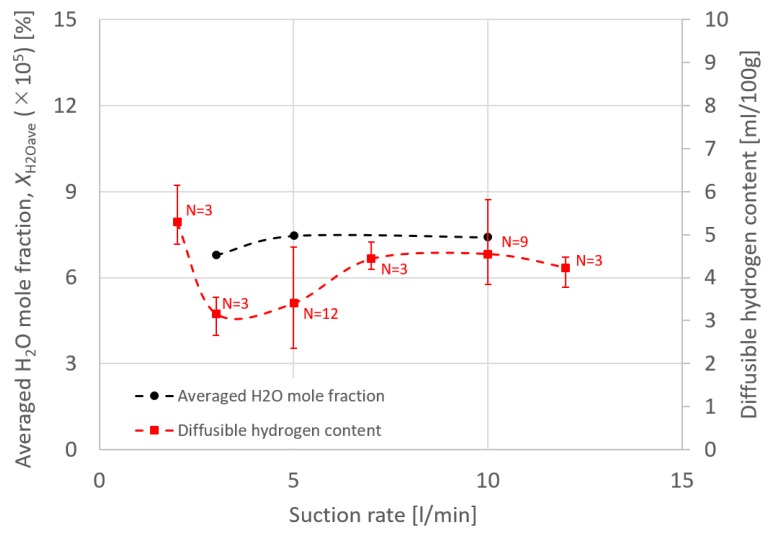
The measured diffusible hydrogen content and the averaged H_2_O mole fraction as a function of the suction rate.

Figure 10, Figure 11, Figure 12 and Figure 13 show the effect of the suction rate. In Figure 10 and Figure 12, the results at 3 and 10 l/min are presented. The gas flow velocity in the vicinity around the suction nozzle largely rose as the suction gas rate increased. In addition, the suction limit height lowered when the suction gas rate was 3 l/min. Figure 12 indicated that even if the higher suction gas rate is set, the higher *η*_sc_ does not obtained. At a suction gas rate of 10 l/min, *η*_sc_ was 48.8% against 59.6% at a suction gas rate of 3 l/min, and thus this leads to a difference of *X*_H2Oave_ between two suction rate cases. The H_2_O in the gas was transported by advection and diffusion, as presented in Equation (5). If diffusion plays an important role in the transport process of H_2_O, the increase in the gas flow velocity in the vicinity around the suction nozzle is thought to increase *η*_sc_. However, such a tendency was not seen in the result. This implies that the advection dominates the transport process. Figure 13 shows the measured diffusible hydrogen content and *X*_H2Oave_ as a function of the suction rate. The diffusible hydrogen content was found to increase with a suction rate above 3 l/min and then become almost constant, which was also in line with the tendency of *X*_H2Oave_. 

Considering the results from Figure 10, Figure 11, Figure 12 and Figure 13 together with the shielding gas flow rate dependency shown in Figure 6, Figure 7, Figure 8 and Figure 9, the relative strength of the shielding gas flow and suction gas flow around the vicinity of the suction nozzle is thought to govern the suction limit height. When the effect of the shielding gas flow is stronger than that of the suction gas, the suction limit height moves to a lower location, leading to a smaller *X*_H2Oave_. This implies that an optimal control of the shielding gas flow rate and suction rate enables reducing the diffusible hydrogen content to some extent.

**Figure 14:**
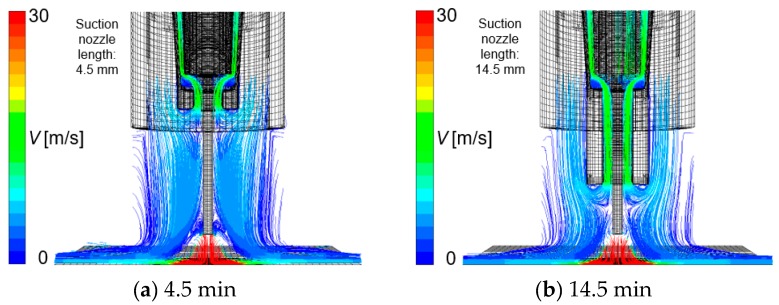
The effect of suction nozzle length on gas flow velocity: (**a**) 4.5 mm, (**b**) 14.5 mm.

**Figure 15:**
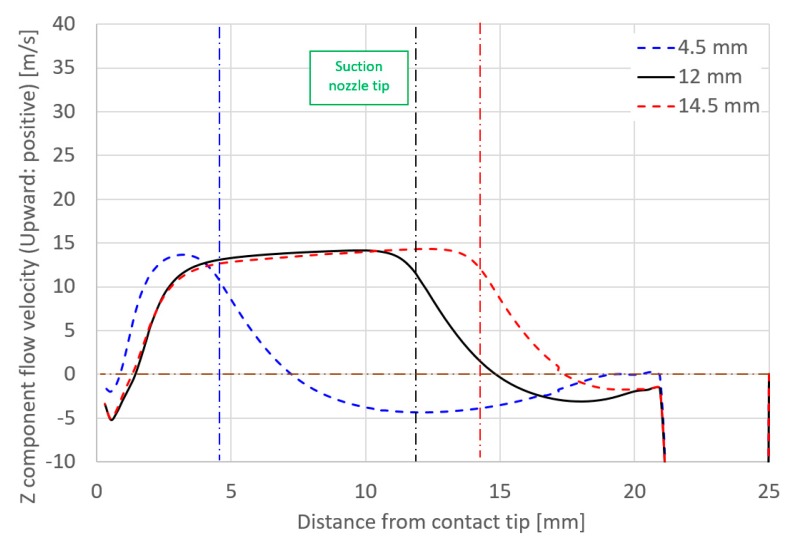
Axial distribution of *Z* component flow velocity as a function of suction nozzle length.

**Figure 16:**
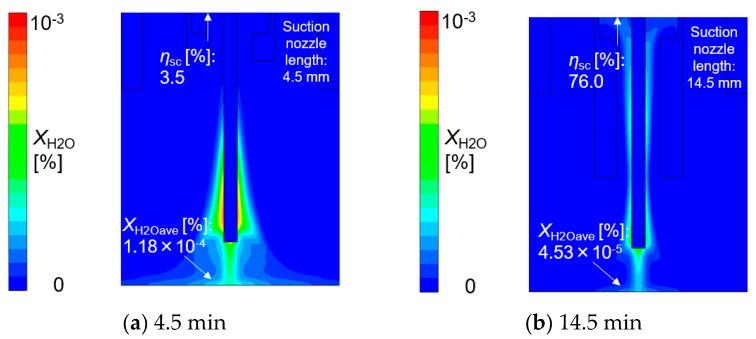
The effect of suction nozzle length on the mole fraction of H_2_O: (**a**) 4.5 mm, (**b**) 14.5 mm.

**Figure 17:**
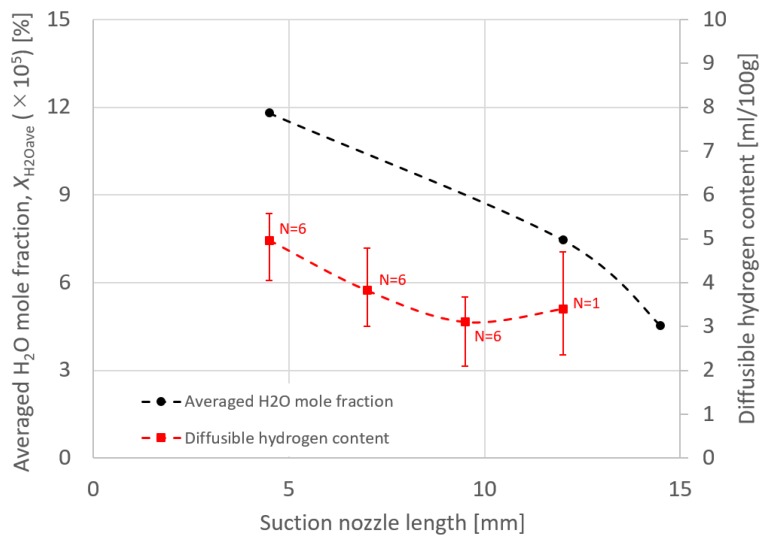
The measured diffusible hydrogen content and the averaged H_2_O mole fraction as a function of suction nozzle length.

Finally, the effect of suction nozzle structure on distributions of the suction gas flow and mole fraction of H_2_O is discussed in this paragraph. Figure 14, Figure 15, Figure 16 and Figure 17 show the effect of the suction nozzle length. In Figure 14 and Figure 16, the results at a suction nozzle length of 4.5 and 14.5 mm are presented. Although the suction limit height greatly lowered with the increase in the suction nozzle length, the relative distance from the suction nozzle tip to the suction limit height was almost a constant value of approximately 3 mm under any conditions. This relative distance was hardly influenced by the suction nozzle length. Figure 17 shows the measured the diffusible hydrogen content and *X*_H2Oave_ as a function of the shielding gas flow rate. It was difficult to carry out the experiment for a suction nozzle length of 14.5 mm, due to the too short distance between the suction nozzle tip and the arc causing the attachment of spatters to the nozzle, and so only experimental results suction nozzle lengths between 4.5 and 12.0 mm were presented. For a nozzle length of 4.5 mm, *η*_sc_ was only 3.5%, which indicated that the suction effect was weak. When the suction nozzle length was extended to 14.5 mm, *η*_sc_ was increased to 76.0% and *X*_H2Oave_ was decreased to 4.53 × 10^−5^%, which was less than half of that in the case of 4.5 mm. As presented in Figure 4, the evaporation rate of H_2_O was predicted to peak at a distance of 15 mm from the contact tip. Accordingly, a suction nozzle length of 14.5 mm is considered to be the most effective at to enable to sucking the H_2_O effectively.

The changes in the H_2_O mole fraction on the weld pool surface in numerical simulation results were compared with the change in the diffusible hydrogen content in experimental results for all the parameters. From the approximate agreement between both the tendencies, the simulation result is considered to almost correctly reflect the behavior of the hydrogen source gas. In this study, only the behavior of the hydrogen source gas in a gas phase was evaluated. However, in the future, the hydrogen diffusion process in deposited metal [30] should be also taken into account in the simulation for direct comparison with the diffusible hydrogen content obtained in the experiment.

Consequently, in order to reduce the diffusible hydrogen content, it was suggested that the suction limit height should be controlled towards below the wire position, the peak of the hydrogen source gas evaporation rate. These two locations can be changed by controlling the suction nozzle design and the torch operating conditions. Furthermore, optimization of the current waveform or adequately designed long suction nozzles have the possibility to decrease the diffusible hydrogen content further. The former is one idea to shift the peak evaporation location upward by enhancing Joule heating of the wire. The latter was found to be particularly effective, but it needs further development of the suction nozzle to prevent spatter attachment.

## 5. Conclusions

In order to reduce the amount of the diffusible hydrogen content in GMAW and FCAW, a novel torch equipped with a dual gas nozzle capable of suctioning the hydrogen source gas desorbed from the wire has been developed. In this paper, the effect of suction nozzle structure and torch operating conditions on suction gas flow pattern and suction efficiency was clarified. The main conclusions are as follows.

(1) Some of the shielding gas introduced from a shielding nozzle was drawn inward and branched into an upward flow that was sucked into the suction nozzle and a downward flow to a base metal. This branching height was defined as the suction limit height, which decisively governed the suction efficiency.
(2) The effect of gas suction from the nozzle on the shielding effect of the welding was shown to be small.
(3) In order to reduce the diffusible hydrogen, it was suggested that the suction limit height should be controlled towards below the wire position, where the evaporation rate of the hydrogen source gas peaks through optimization of the suction nozzle design and the torch operating conditions.
(4) The relative relationship between shielding gas flow and suction gas flow around the vicinity of the suction nozzle were thought to govern the suction limit height. When the effect of the shielding gas flow was stronger than that of the suction gas, the suction limit height moved to a lower location, leading to a smaller *X*_H2Oave_.
(5) This method was suggested to be particularly effective for reducing the diffusible hydrogen content to extend the suction nozzle length. However, further development to prevent spatter attachment to the nozzle is required.

## Figures and Tables

**Table 1:** Simulation conditions.

Region Size	40.0 mm(X) × 20.0 mm(Y) × 56.5 mm(Z)
Shielding gas	CO_2_
Shielding gas flow rate	15, 25, or 35 l/min (25 l/min is the standard condition)
Suction nozzle length	4.5, 12, or 14.5 mm (12 mm is the standard condition)
Suction nozzle diameter	4 mm
Suction rate	3, 5, or 10 l/min (5 l/min is the standard condition)
Contact tip-to-work distance (CTWD)	25 mm
Arc length	4 mm
Welding current	DC280 A

**Table 2:** Boundary conditions.

Boundary		Velocity	Mass Fraction of CO_2_	Mass Fraction of H_2_O	Energy	Electric Potential	Vector Potential
Top	Shielding gas inlet	$\vec{u}$ = $\vec{u}$_shield_	*Y_CO2_* = 1	*Y_H2O_* = 0	300 K	*∂Φ*/*∂n* = 0	*∂A*_i_/*∂n* = 0
	Suction gas outlet	$\vec{u}$ = $\vec{u}$_suction_	*∂Y_CO2_*/*∂n* = 0	*∂Y_H2O_*/*∂n* = 0	300 K	*∂Φ*/*∂n* = 0	*∂A*_i_/*∂n* = 0
	Nozzles	−	−	−	300 K	*∂Φ*/*∂n* = 0	*∂A*_i_/*∂n* = 0
	Contact tip	−	−	−	300 K	*σ∂Φ*/*∂n* = *j*_given_	*∂A*_i_/*∂n* = 0
	Gas	*P* = 0	*Y_CO2_* = 0 (inflow) *Y_CO2_*/*∂n* = 0 (outflow)	*Y_H2O_* = 0 (inflow) *Y_H2O_*/*∂n* = 0 (outflow)	300 K	*∂Φ*/*∂n* = 0	*∂A*_i_/*∂n* = 0
Side	Gas	*P* = 0	*Y_CO2_* = 0 (inflow) *Y_CO2_*/*∂n* = 0 (outflow)	*Y_H2O_* = 0 (inflow) *Y_H2O_*/*∂n* = 0 (outflow)	300 K	*∂Φ*/*∂n* = 0	*A*i = 0
Bottom	Base metal	$\vec{u}$ = 0	*∂Y_CO2_*/*∂n* = 0	*∂Y_H2O_*/*∂n* = 0	300 K	*Φ* = 0	*∂A*_i_/*∂n* = 0
